# Rapid visual detection of hepatitis E virus combining reverse transcription recombinase-aided amplification with lateral flow dipstick and real-time fluorescence

**DOI:** 10.1128/jcm.01064-24

**Published:** 2025-01-16

**Authors:** Bingyan Wei, Wenlong Wang, Zixuan Guo, Wenjiao Yin, Minheng Cheng, Yifei Yang, Yuewei Tian, Yaxin Sun, Tianlong Liu, Yanxin Hu, Ruiping She, Jijing Tian

**Affiliations:** 1 Laboratory of Animal Pathology and Public Health, National Key Laboratory of Veterinary Public Health and Safety, College of Veterinary Medicine, China Agricultural University 630101 , Beijing, China; 2 School of Basic Medical Sciences, Xi'An Jiaotong University 598900 , Xi'An, Shaanxi, China; 3 National Institute for Viral Disease Control and Prevention, Chinese Center for Disease Control and Prevention 12415 , Beijing, China; 4 Beijing Center for Animal Disease Control and Prevention, Beijing, China; University of California, Davis, Davis, California, USA

**Keywords:** hepatitis E virus, lateral flow dipstick, nucleic acid detection, recombinase-aided amplification

## Abstract

Hepatitis E virus (HEV) is a globally prevalent zoonotic pathogen that is primarily spread through the fecal-oral route, such as by consuming undercooked or contaminated pork. HEV infection leads to an estimated 3.3 million symptomatic cases of viral hepatitis and 70,000 deaths in human populations each year. Therefore, a rapid and accurate method for detecting HEV in serum or stool samples is essential. In this study, we aimed to develop and evaluate two methods for the rapid and convenient detection of HEV RNA: reverse transcription recombinase-aided amplification with lateral flow dipstick (RT-RAA-LFD) and quantitative real-time reverse transcription recombinase-aided amplification (qRT-RAA). We optimized the reaction conditions and assessed their sensitivity and specificity. The RT-RAA-LFD assay completed its reaction at 39°C within 15 minutes, achieving a 95% limit of detection (LOD) of 247 copies/μL. The qRT-RAA assay, completed at 42°C within 20 minutes, had a 95% LOD of 25 copies/μL. Both assays demonstrated no cross-reactivity with other porcine pathogens and exhibited strong specificity. In testing 245 porcine bile and fecal samples, the RT-RAA-LFD assay showed a kappa value of 0.943 (*P* < 0.001) with a 97.14% (238/245) coincidence rate compared with quantitative reverse transcription PCR. Similarly, the qRT-RAA assay achieved a kappa value of 0.976 (*P* < 0.001) with a 98.78% (242/245) coincidence rate. In conclusion, these two RT-RAA assays show promising potential as effective diagnostic tools for broad and efficient screening of swine HEV in veterinary clinics.

**IMPORTANCE:**

Hepatitis E virus (HEV) is a globally widespread zoonotic pathogen that poses a significant public health risk. Swine serve as the primary natural host for zoonotic HEV. This study introduces a rapid and precise method for detecting swine HEV RNA, showcasing its potential as an effective diagnostic tool for comprehensive and efficient screening of swine HEV in veterinary clinics.

## INTRODUCTION

Hepatitis E virus (HEV) is a widespread cause of acute viral hepatitis, posing significant global public health challenges. Swine are one of the most important natural reservoir hosts of HEV. Currently, four HEV genotypes (HEV-3–HEV-6) have been identified in both domestic and wild boar populations. Among these, HEV-3 and HEV-4 are zoonotic pathogens capable of infecting both humans and non-human primates, whereas HEV-5 and HEV-6 have been found exclusively in wild boar populations (1). HEV-3 predominantly circulates in Europe, Asia, and America, while HEV-4 is primarily found in Europe and Asia (2). Research has shown notable differences in HEV infection rates among domestic pigs in different countries or regions. The seroprevalence rate for HEV IgG antibodies varied between approximately 30% to 98%, while the positivity rate for HEV RNA ranged from about 10% to 100% (3). These findings indicate that the prevention and control of HEV have significant public health implications.

HEV is transmitted primarily via the fecal-oral route (4). The virus replicates in hepatocytes and bile duct epithelial cells. Once released into the bile duct lumen, it is excreted through the intestinal tract and eliminated from the body via feces (5, 6). Consumption of undercooked or raw contaminated pork or pig liver is considered a primary route for HEV transmission from swine to humans (7). Sporadic cases of HEV infection are frequently linked to the consumption of undercooked animal products, particularly pork and wild boar meat (8). Workers in swine farms and slaughterhouses face a relatively high risk of zoonotic HEV infection (9). In the majority of healthy individuals, acute hepatitis infection is typically self-limiting and asymptomatic and either resolves spontaneously or heals after mild gastrointestinal symptoms (10). High-risk groups for severe HEV infection and potential fatal outcomes include infants, the elderly, pregnant women, immunocompromised individuals, patients with concurrent disorders, and workers frequently exposed to HEV-infected animals (11). Among immunocompromised individuals, acute HEV infection can progress to severe fulminant hepatitis (acute liver failure), chronic hepatitis, or even cirrhosis (12, 13). Besides liver diseases, some patients may exhibit extrahepatic manifestations, including neurological, hematologic, and kidney disorders (14). Therefore, rapid and reliable diagnosis and monitoring of HEV are crucial to ensure both the safety of animal-source foods and public health.

**Fig 1:**
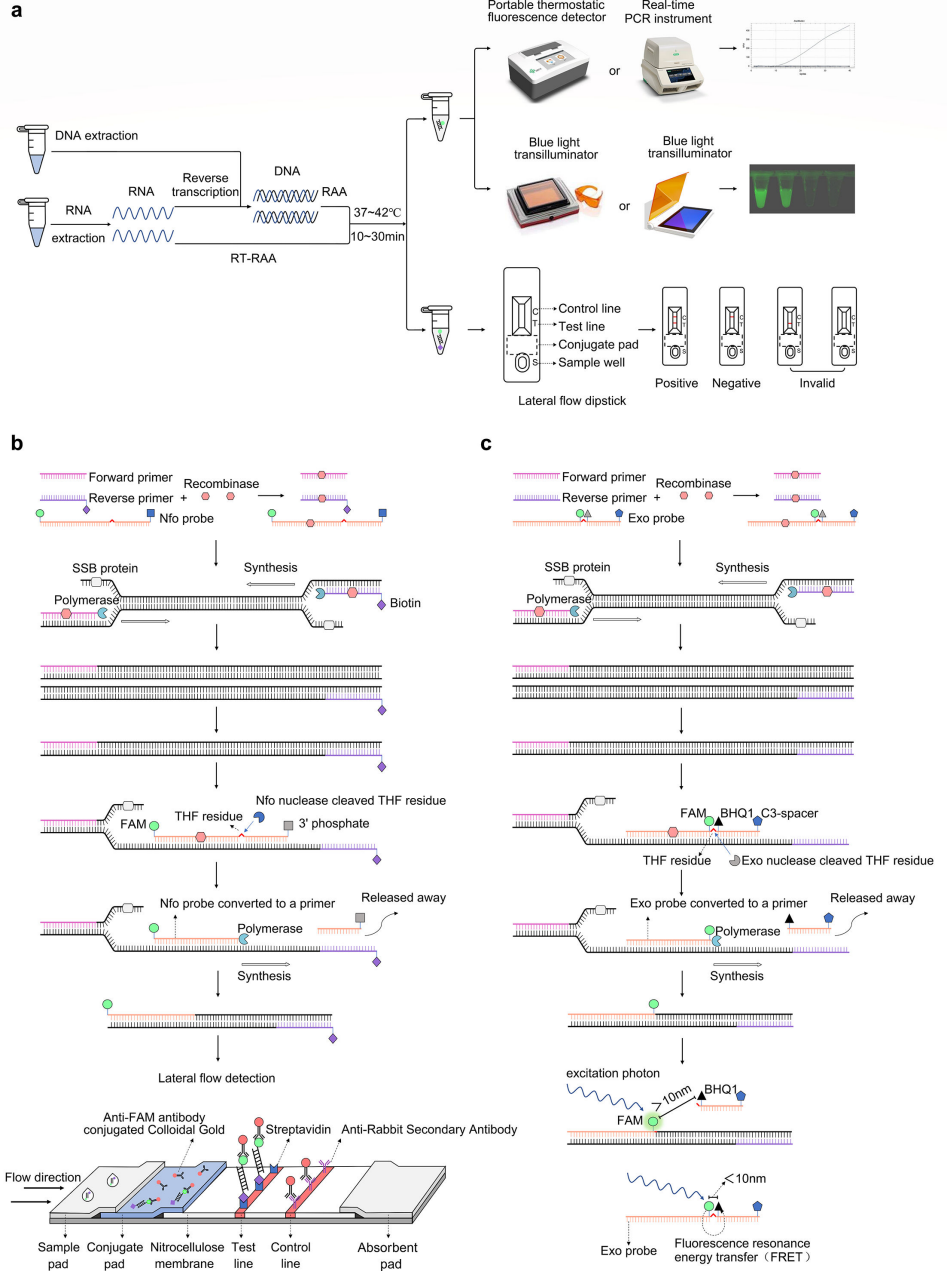
Illustration of reverse transcription recombinase-aided amplification with lateral flow dipstick (RT-RAA-LFD) and quantitative real-time reverse transcription recombinase-aided amplification (qRT-RAA) reaction. (**a**) Flowchart of the RT-RAA-LFD and qRT-RAA reactions. (**b**) Schematic representation of the RT-RAA-LFD reaction. The recombinase forms complexes with the primer or probe. The recombinase-primer complex then recognizes and binds to a homologous sequence on the DNA template. Simultaneously, the SSBs stabilize the complementary strand in its single-stranded state. Following this, the recombinase dissociates from the primer and makes it accessible for DNA polymerase to bind to the 3′ end of the primer. This binding initiates a strand displacement reaction and allows rapid amplification of a specific DNA fragment. The amplification product serves as a new template for hybridization with a nfo-probe under the action of recombinase. Endonuclease IV (nfo) cleaves the probe at the tetrahydrofuran (THF) residue and creates a new 3′ end that serves as the initiation site for DNA polymerase to synthesize a new DNA strand. This cleavage transforms the probe into a primer. The final amplification product is labeled with antigenic labels at the 5′ ends of both the probe and primer, with 6-carboxyfluorescein (FAM) and biotin, respectively. The doubly labeled amplification product then binds to colloidal gold-labeled anti-FAM antibodies on the conjugate pad as it flows through the sample pad. Subsequently, it flows through the detection area (T line), where it is captured by streptavidin immobilized in the line. The formation of sandwich structure on the T line exhibits a red band. Finally, any excess colloidal gold-labeled anti-FAM antibodies are captured by anti-rabbit secondary antibodies immobilized on the quality control zone (C line), leading to the appearance of a red band on the C line. (**c**) Schematic representation of the qRT-RAA reaction. The forward and reverse primers bind to the template, initiating the amplification process. The amplification product serves as a new template for further amplification. In the next step, the exo probe hybridizes to the target sequence with the assistance of recombinase. Once hybridized, exonuclease III (exo) cleaves the probe at the THF residue. This cleavage event results in the spatial separation of the fluorescent group (FAM) from the quenching group 1 (BHQ1). Under stimulation by incident light, fluorescence resonance energy transfer does not occur and FAM emits fluorescence. The fluorescence intensity is directly proportional to the accumulation of amplification products as the amplification progresses. Thus, the assay enables real-time monitoring of the amplification products.

Several molecular biological techniques, including reverse transcription-nested PCR (RT-nPCR), quantitative reverse transcription PCR (qRT-PCR), and loop-mediated isothermal amplification (LAMP), are established for HEV nucleic acid detection (15). Among these methods, TaqMan-based real-time RT-PCR is most commonly used for HEV detection. This method, although highly sensitive and accurate, requires an accurate and stable thermal cycler, as well as a reliable power supply circuit. Consequently, it is limited to well-equipped laboratories (16). Thus, it is imperative to develop user-friendly, portable, visually interpretable methods with high sensitivity and specificity for on-site detection of HEV to enhance disease prevention and control. Recombinase polymerase amplification (RPA) and recombinase-aided amplification (RAA) are rapid and highly specific techniques for pathogen detection (17). The RPA/RAA reaction utilizes three core enzymes: recombinase, single-stranded DNA-binding protein (SSB), and DNA polymerase. Recombinase recognizes the target sequence, SSB stabilizes the reaction, and DNA polymerase amplifies the specific DNA segment (18). The difference between RPA and RAA is that RPA utilizes bacteriophage T4 recombinase, whereas RAA employs recombinase derived from bacteria or fungi (19). These reactions occur within 15–30 minutes at 37–42°C. Detection can be performed through agarose gel electrophoresis, lateral flow dipstick (LFD), or real-time fluorescence. The procedure and principle of the RAA reaction are depicted in Fig. 1.

In the present study, we aim to establish a reverse transcription recombinase-aided amplification (RT-RAA) technique combined with LFD and real-time fluorescence for the rapid and convenient detection of HEV in swine. Through comparative analysis with qRT-PCR, we evaluated the specificity, sensitivity, and detection capabilities of RT-RAA. Our findings demonstrate that RT-RAA is an effective method for detecting HEV RNA in swine populations, suitable for use in less-equipped laboratories or resource-constrained regions.

## MATERIALS AND METHODS

### Viruses and clinical samples

The HEV-positive fecal sample (GenBank accession no. MZ751061) was generously provided by Professor Chunnan Liang from the China National Institutes for Food and Drug Control. The cDNA or viral DNA samples of swine influenza virus (H1N1), porcine reproductive and respiratory syndrome virus (PRRSV), African swine fever virus (ASFV), and porcine circovirus 2 (PCV2) were obtained from the epidemiological investigations conducted by the Animal Disease Prevention and Control Center, Beijing, China. A total of 100 fresh gallbladders were collected from a pig slaughterhouse located in the suburbs of Beijing. Additionally, 52 bile samples and 93 fecal samples were epidemiological survey specimens preserved in the laboratory and identified as HEV-positive or HEV-negative using qRT-PCR.

### Nucleic acid extraction

Bile samples from the gallbladders were mixed with PBS and centrifuged at 12,000 rpm for 15 minutes at 4°C. Total RNA from bile supernatants and fecal samples were extracted using a TRIzol-based method according to the manufacturer’s protocol (Thermo Fisher Scientific, Waltham, MA, USA). The extracted RNA was reverse transcribed into cDNA and stored at −20°C.

### Preparation of standard recombinant plasmids

The ORF2 gene from the HEV-3 KernowC1/p6 strain (GenBank accession no. HQ709170.1) was synthesized by Sangon Biotech Co. Ltd. (Shanghai, China) and ligated into the pUC57 vector, resulting in the recombinant plasmid designated as pUC57-ORF2. To convert DNA concentration to copy number in the standard plasmid solution, the following formula was employed:


$$Plasmid\;copy\;number\;\left(copies/\mu L\right)=\frac{6.02\times 10^{14}\times plasmid\;concentration\;\left(ng/\mu L\right)}{660\times vector\;and\;target\;fragment\;length\;\left(bp\right)}$$

### Primer and probe design and synthesis

To screen target regions of primers and probes designed for HEV detection, 62 genomic sequences of HEV-3 strains and 62 genomic sequences of HEV-4 strains were downloaded from the GenBank nucleic acid sequence database on NCBI (Fig. S1). These sequences were aligned using MEGA X software. Following primer and probe design principles from the twistAmp RPA manual, we developed a comprehensive set of molecular tools, including 1 test strip probe (nfo-probe), 1 fluorescent probe (exo-probe), 7 forward primers (F1**–**F7), and 7 reverse primers (R1–R7) for the primary screening and 22 forward primers (F6-1–F7-10) and 17 reverse primers (R3-1–R3-16) for the secondary screening. These tools were designed in a conserved region using SnapGene 6.1.1 with details listed in Table 1.

**TABLE 1:** The primers and probes of the HEV-based RT-RAA-LFD, qRT-RAA, qRT-PCR assays

Primers/probes	Nucleotide sequences (5′→3′)	Primer/probe position^*a*^
F1	TTCATTTTACTGGGACTAATGGCGTTGGTG	6416–6445
F2	CTAATGGCGTTGGTGAGGTGGGTCGTGGTA	6431–6460
F3	AGGTGGGTCGTGGTATAGCGCTGACACTGT	6446–6475
F4	TAGCGCTGACACTGTTTAATCTTGCTGACA	6461–6490
F5	TTAATCTTGCTGACACGCTTCTCGGTGGGC	6476–6505
F6	CGCTTCTCGGTGGGCTGCCGACAGAATTGA	6491–6520
F7	TGCCGACAGAATTGATTTCGTCGGCTGGGG	6506–6535
F6-1	ACACGCTTCTCGGTGGGCTGCCGACAGAAT	6488–6517
F6-2	CACGCTTCTCGGTGGGCTGCCGACAGAATT	6489–6518
F6-3	ACGCTTCTCGGTGGGCTGCCGACAGAATTG	6490–6519
F6-4	GCTTCTCGGTGGGCTGCCGACAGAATTGAT	6492–6521
F6-5	CTTCTCGGTGGGCTGCCGACAGAATTGATT	6493–6522
F6-6	TTCTCGGTGGGCTGCCGACAGAATTGATTT	6494–6523
F6-7	TCTCGGTGGGCTGCCGACAGAATTGATTTC	6495–6524
F6-8	CTCGGTGGGCTGCCGACAGAATTGATTTCG	6496–6525
F6-9	TCGGTGGGCTGCCGACAGAATTGATTTCGT	6497–6526
F6-10	CGGTGGGCTGCCGACAGAATTGATTTCGTC	6498–6527
F7-1	GGTGGGCTGCCGACAGAATTGATTTCGTCG	6499–6528
F7-2	GTGGGCTGCCGACAGAATTGATTTCGTCGG	6500–6529
F7-3	TGGGCTGCCGACAGAATTGATTTCGTCGGC	6501–6530
F7-4	GGGCTGCCGACAGAATTGATTTCGTCGGCT	6502–6531
F7-5	GTTTGCCGACAGAATTGATTTCGTCGGCTG	6503–6532
F7-6	TTTGCCGACAGAATTGATTTCGTCGGCTGG	6504–6533
F7-7	TTGCCGACAGAATTGATTTCGTCGGCTGGG	6505–6534
F7-8	GCCGACAGAATTGATTTCGTCGGCTGGGGG	6507–6536
F7-9	CCGACAGAATTGATTTCGTCGGCTGGGGGT	6508–6537
F7-10	CGACAGAATTGATTTCGTCGGCTGGGGGTC	6509–6538
R1	CATTCTCGACTGATGTGTAAAGCTTCACTG	6584–6613
R2	CCTTATCCTGCTGCGCATTCTCGACTGATG	6599–6628
R3	GTGGGATAGCAATACCCTTATCCTGCTGCG	6614–6643
R4	CAAGATCAATATCGTGTGGGATAGCAATAC	6629–6658
R5	CAACACGGGACTCACCAAGATCAATATCGT	6644–6673
R6	CATAATCCTGGATAACAACACGGGACTCAC	6659–6688
R7	GCTCGTGCTGGTTATCATAATCCTGGATAA	6673–6703
R3-1	GCAATACCCTTATCCTGCTGCGCATTCTCG	6606–6635
R3-2	AGCAATACCCTTATCCTGCTGCGCATTCTC	6607–6636
R3-3	TAGCAATACCCTTATCCTGCTGCGCATTCT	6608–6637
R3-4	ATAGCAATACCCTTATCCTGCTGCGCATTC	6609–6638
R3-5	GATAGCAATACCCTTATCCTGCTGCGCATT	6610–6639
R3-6	GGATAGCAATACCCTTATCCTGCTGCGCAT	6611–6640
R3-7	GGGATAGCAATACCCTTATCCTGCTGCGCA	6612–6641
R3-8	TGGGATAGCAATACCCTTATCCTGCTGCGC	6613–6642
R3-9	TGTGGGATAGCAATACCCTTATCCTGCTGC	6615–6644
R3-10	GTGTGGGATAGCAATACCCTTATCCTGCTG	6616–6645
R3-11	CGTGTGGGATAGCAATACCCTTATCCTGCT	6617–6646
R3-12	TCGTGTGGGATAGCAATACCCTTATCCTGC	6618–6647
R3-13	ATCGTGTGGGATAGCAATACCCTTATCCTG	6619–6648
R3-14	TATCGTGTGGGATAGCAATACCCTTATCCT	6620–6649
R3-15	ATATCGTGTGGGATAGCAATACCCTTATCT	6621–6650
R3-16	AATATCGTGTGGGATAGCAATACCCTTATC	6622–6651
Final F	TGGGYTGCCGACAGAATTGATTTCGTCGGC	6501–6530
Final Rn	Biotin-AGYAATACCCTTRTCCTGCTGCGCATTCTC	6607–6636
Final Re	AGYAATACCCTTRTCCTGCTGCGCATTCTC	6607–6636
nfo-probe	FAM-TGTTTTACTCYCGCCCCGTTGTCTCRGCCA [THF]TGGCGAGCCGACAGT-phosphorylation	6542–6587
exo-probe	TTGTCTCRGCCAATGGCGAGCCGACWG(FAM-dT)GA [THF]AC(BHQ1-dT)TTACACATCAGT[C3-spacer]	6561–6606
HEV-F	GGTGGTTTCTGGGGTGAC	5473–5490
HEV-R	AGGGGTTGGTTGGATGAA	5525–5542
HEV-P	FAM-TGATTCTCAGCCCTTCGC-BHQ	5496–5513

^*a*^ The positions of probes and primers are referenced to the genomic sequence of the Kernow-C1 strain (GenBank accession no. HQ709170.1). FAM, 6-carboxyfluorescein; THF, tetrahydrofuran; BHQ1, black hole quencher 1.

For reverse transcription recombinase-aided amplification with lateral flow dipstick (RT-RAA-LFD), the nfo-probe was modified with a fluorescent group (FAM) at its 5ʹ end and a polymerase extension blocking group (phosphate) at its 3ʹ end. At position 31, the A residue was substituted with a tetrahydrofuran (THF) residue. The final reverse primer (Final Rn) of RT-RAA-LFD was labeled with biotin at its 5ʹ end. For quantitative real-time reverse transcription recombinase-aided amplification (qRT-RAA), the 3ʹ end of the exo-probe was labeled with a polymerase extension blocking group (C3-spacer). Specifically, at positions 28, 31, and 34 of the exo-probe, the nucleotides T, A, and T were substituted with distinct chemical groups: a dT-fluorophore group (FAM-dT), a THF residue, and a dT-quencher group (BHQ1-dT), respectively. All the primers and probes used in the study were synthesized by Sangon Biotech Co. Ltd. (Shanghai, China).

### Agarose gel electrophoresis-based RT-RAA assay

A 25 µL reaction mixture was prepared according to the instructions of the basic DNA thermostatic rapid amplification kit (Amplification Future Biotech Co. Ltd., Weifang, China). The reaction mixture contained 14.7 µL buffer A, 4.55 µL ddH_2_O, 1 µL forward primers (10 µM), 1 µL reverse primers (10 µM), 2.5 µL DNA template, and 1.25 µL buffer B. The tubes containing the reaction mixture were briefly vortexed, centrifuged, and then placed in the PCR instrument, where they were incubated for 30 minutes at 39°C. After amplification, the RAA products were combined with 5 µL of 6× loading buffer, thoroughly mixed, and analyzed through a 3% agarose gel electrophoresis.

### RT-RAA-LFD and qRT-RAA assay

For the RT-RAA-LFD assay, a 25 µL reaction mixture was prepared according to the manufacturer’s instructions for the colloidal gold strip-type DNA thermostatic rapid amplification kit (Amplification Future Biotech Co. Ltd., Weifang, China). The reaction mixture contained 14.7 µL buffer A, 4.25 µL ddH_2_O, 1 µL forward primers (10 µM), 1 µL reverse primers (10 µM), 2.5 µL DNA template, 0.3 µL nfo-probes (10 µM), and 1.25 µL buffer B. The reaction tubes were briefly vortexed, centrifuged, and then placed in the T100 PCR thermal cycler (Bio-Rad, Hercules, CA, USA), where they were incubated for 15 minutes at 39°C. After amplification, the resulting products were diluted 10-fold with ddH_2_O. Subsequently, 100 µL of the diluted reaction mixture was added to the sample wells, and observations were made over a 5-minute period. A positive result was indicated by the appearance of red visible bands in both the quality control area (C line) and the detection area (T line) of the LFD. Conversely, a negative result was indicated by the presence of a red band only in the quality control area. An invalid result was reported if no red band was observed in the quality control area.

For the qRT-RAA assay, a 25 µL reaction mixture was prepared following the instructions provided by the real-time fluorescent-type DNA thermostatic rapid amplification kit (Amplification Future Biotech Co. Ltd., Weifang, China), identical to RT-RAA-LFD. The reaction tubes were briefly vortexed and then centrifuged before being placed in the CFX96 Bio-Rad Real-Time PCR Thermal Cycler (Bio-Rad, Hercules, CA, USA). They were then incubated for 20 minutes at 42°C, with data collected in the FAM channel every 30 seconds. Samples were classified as positive if their amplification curve exceeded the threshold of the negative control exponentially. After the reaction was completed, the amplification products were observed under excitation light to detect the green fluorescence emitted by positive amplification.

### qRT-PCR assay

The primer pair (HEV-F/R) and TaqMan probe (HEV-P) used in the qRT-PCR were adopted from the previous study (20). A 20 µL reaction mixture was prepared according to the manufacturer’s instructions for the FastFire quantitative PCR premix (Tiangen Biotech Co. Ltd., Beijing, China), comprising 10 µL 2× FastFire qPCR premix, 7.4 µL ddH_2_O, 0.6 µL forward primers (10 µM), 0.6 µL reverse primers (10 µM), 0.4 µL probes (10 µM), and 1 µL DNA template. Amplification was conducted using the CFX96 Bio-Rad Real-Time PCR Thermal Cycler (Bio-Rad, Hercules, CA, USA) with the following thermal cycling program: 95°C for 1 minute, 40 cycles of 95°C for 5 seconds, and 60°C for 15 seconds.

### Optimization of the RT-RAA assay reaction conditions

The efficiency of amplification in the RT-RAA assay could be influenced by the reaction conditions. Using 2.9 × 10^5^ copies/μL pUC57-ORF2 recombinant plasmid as a template, we optimized the reaction temperature and time with the optimal forward primer, reverse primer, and probes. To determine the optimal reaction temperature of the RT-RAA-LFD assay, a series of experiments were conducted with reaction temperatures of 30°C, 33°C, 36°C, 39°C, and 42°C, maintaining a constant reaction time of 20 minutes. At the optimal reaction temperature, various reaction times of 0, 5, 10, 15, and 20 minutes were tested to determine the most suitable reaction time. The optimal reaction temperature and time of the RT-RAA-LFD assay were determined by the extent of T-line color development. To optimize the qRT-RAA assay reaction conditions, the reaction temperatures were precisely controlled at 36°C, 38°C, 40°C, and 42°C, with a consistent reaction time of 25 minutes. The optimal reaction temperature of the qRT-RAA assay was determined by both fluorescence intensity and peak appearance time.

### Specificity analysis

Under optimal reaction conditions of both the RT-RAA-LFD and qRT-RAA assays, nucleic acids from HEV, H1N1, PRRSV, ASFV, and PCV2 were used as templates to assess the specificity of the primers and probes employed in two assays.

### Sensitivity analysis

The pUC57-ORF2 recombinant plasmid underwent a 10-fold serial dilution to obtain plasmid concentrations ranging from 2.9 × 10^5^ to 2.9 × 10^−1^ copies/μL. Each plasmid concentration was subjected to three independent detections using RT-RAA-LFD, qRT-RAA, and qRT-PCR assays, conducted under optimal reaction conditions. The detection results were analyzed using Probit regression analysis with SPSS 27.0 software to determine the 95% limit of detection (95% LOD) and were compared with the standard detection method, qRT-PCR.

### Evaluation of diagnostic performance

We employed the RT-RAA-LFD, qRT-RAA, and qRT-PCR assays to analyze 245 porcine bile and fecal samples. The results were compared using the Kappa statistic to assess the clinical utility of the RT-RAA and RT-RAA-LFD assays. The concordance of clinical diagnostic performance between the RT-RAA and qRT-PCR assays was evaluated using the Kappa statistic with SPSS 27.0 software. A kappa value of 1.000 denoted perfect agreement. A *P* value < 0.05 indicated a statistically significant difference.

## RESULTS

### Screening of optimal primer-probe combinations for the RT-RAA assays

**Fig 2:**
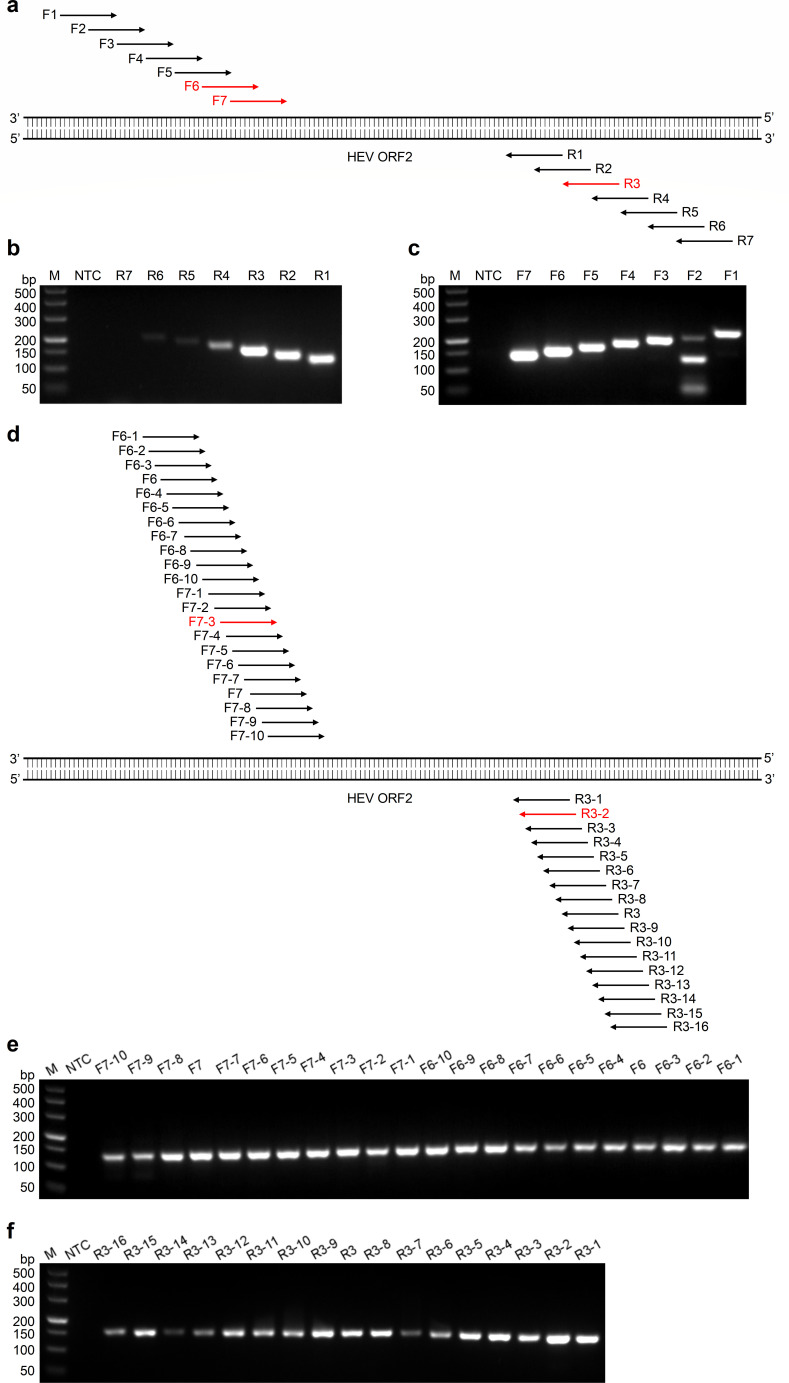
Screening of the optimal primer pair for the RT-RAA assays. (**a**) Schematic diagram of primary primer screening. Seven forward primers (F1–F7) and seven reverse primers (R1–R7) are indicated by arrows, with their relative positions in the HEV ORF2 gene. (**b**) Primary screening results analyzed by agarose gel electrophoresis for the optimal reverse primer. RT-RAA reactions were conducted by utilizing F6 to screen all the reverse primers (R1–R7). (**c**) Primary screening results analyzed by agarose gel electrophoresis for the optimal forward primer. RT-RAA reactions were conducted by utilizing R3 to screen all the forward primers (F1–F7). (**d**) Schematic diagram of secondary primer screening. Twenty-two forward primers (F6-1–F7-10) and 17 reverse primers (R3-1–R3-16) are indicated by arrows, with their relative positions in the HEV ORF2 gene. (**e**) Secondary screening results analyzed by agarose gel electrophoresis for the optimal forward primer. RT-RAA reactions were conducted by utilizing R3 to screen all the forward primers (F6-1–F7-10). (**f**) Secondary screening results analyzed by agarose gel electrophoresis for the optimal reverse primer. RT-RAA reactions were conducted by utilizing F7-3 to screen all the reverse primers (R3-1–R3-16). NTC, no template control; M, DL500 DNA marker.

We aligned genomic sequences from 124 global HEV strains and designed seven forward primers (F1–F7) and seven reverse primers (R1–R7) targeting conserved regions (Fig. 2a). Among these primers, F6 was arbitrarily selected to screen all reverse primers. After agarose gel electrophoresis, the amplification product of R3 exhibited the brightest band, indicating the most efficient amplification (Fig. 2b). Therefore, R3 was chosen to screen all forward primers. To quantitatively evaluate the primer pair performance, we utilized Image Lab 3.0 software and found that the bands of F6 and F7 exhibited similar brightness (Fig. 2c). Consequently, the primer pair F6/F7 and R3 emerged as the most suitable combination for the primary screening.

To enhance the detection performance of RT-RAA assays, we employed a strategy to design the primers of the secondary screening. This strategy involved maintaining the primer length unchanged while increasing their quantity by systematically adjusting their relative positions in the template, moving forward or backward at the speed of one base to refine the primary primer pair. We designed 22 forward primers (F6-1–F7-10) and 17 reverse primers (R3-1–R3-16). Due to the similar brightness in the bands of F6 and F7, we ensured that the sequence of F6-1 covered the first three bases at the 5′ end of F6 and the sequence of F7-10 covered the last three bases at the 3′ end of F7 (Fig. 2d). The second screening was conducted by utilizing the top-performing reverse primer R3 from the primary screening to screen all forward primers, followed by analysis of the amplification products through agarose gel electrophoresis. F7-3 showed the most optimal amplification performance, and it was chosen to screen all reverse primers (Fig. 2e). Similarly, the intensity of the brightest band corresponded to R3-2 (Fig. 2f). Therefore, the most suitable primer combination for the secondary screening was the primer pair F7-3 and R3-2.

Ultimately, degenerate bases were incorporated into the final primer pair and probes to minimize the occurrence of mismatched bases. Final F, Final Rn, and nfo-probes were used for the RT-RAA-LFD assay, while Final F, Final Re, and exo-probe were utilized for the qRT-RAA assay. The positions of the RT-RAA primer pair, nfo-probe, and exo-probe in the ORF2 gene are shown in Fig. S1.

### Optimization of the RT-RAA assay reaction conditions

**Fig 3:**
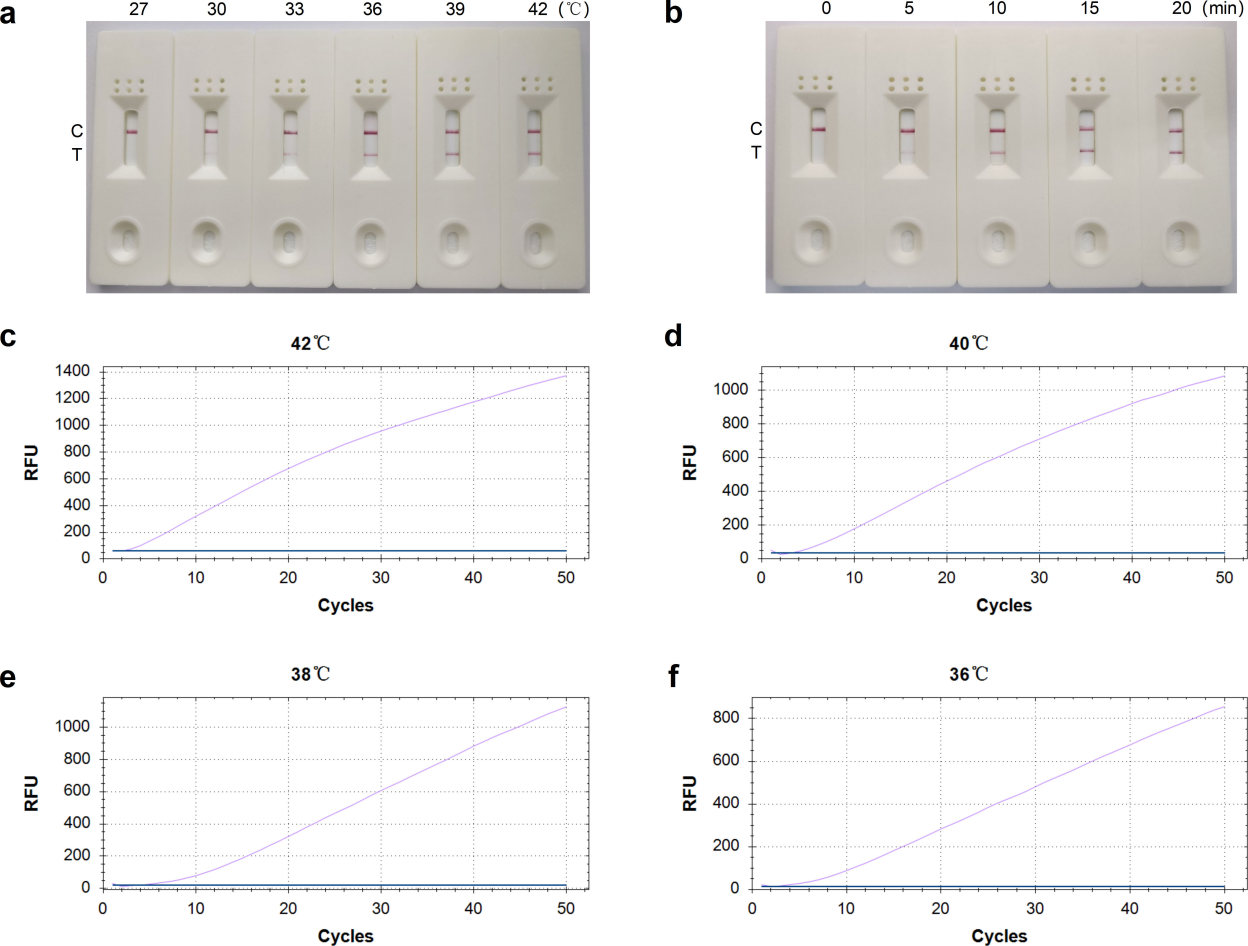
Optimization of the RT-RAA-LFD and qRT-RAA assay reaction conditions for HEV detection. (a) Reaction temperature optimization of the RT-RAA-LFD assay. The reaction mixtures were incubated at 30°C, 33°C, 36°C, 39°C, and 42°C for 20 minutes, and the amplification products were visualized using LFD. (b) Reaction time optimization of the RT-RAA-LFD assay. The reaction mixtures were incubated at 39°C for 0, 5, 10, 15, and 20 minutes, and the amplification products were visualized using LFD. (c–f) Reaction temperature optimization of the qRT-RAA assay. The reaction mixtures were incubated at 36°C, 38°C, 40°C, and 42°C for 25 minutes, with data collected in the FAM channel every 30 seconds using the real-time PCR instrument.

Using 2.9 × 10^5^ copies/μL of pUC57-ORF2 recombinant plasmid standard as a template, the optimal forward and reverse primers and probes were utilized to determine the optimal reaction conditions for RT-RAA-LFD and qRT-RAA. Figure 3a shows that the test yielded a negative result at 27°C, with a faint T line visible at 30°C. As the temperature increased, the intensity of the T line’s color gradually increased, with a distinct T line appearing at 39°C and 42°C. Therefore, 39°C was determined to be the optimal reaction temperature. Figure 3b shows a weak T line at 5 minutes and 10 minutes, which became clearer at 15 minutes and 20 minutes. Therefore, 15 minutes was confirmed as the optimal reaction time. In summary, the optimal reaction condition for RT-RAA-LFD was 39°C for 15 minutes. As shown in Fig. 3c through f, the amplification curve at 42°C achieved the highest fluorescence signal and the earliest peak appearance time, thus confirming 42°C as the optimal qRT-RAA reaction temperature.

### Specificity analysis of the RT-RAA assays

**Fig 4:**
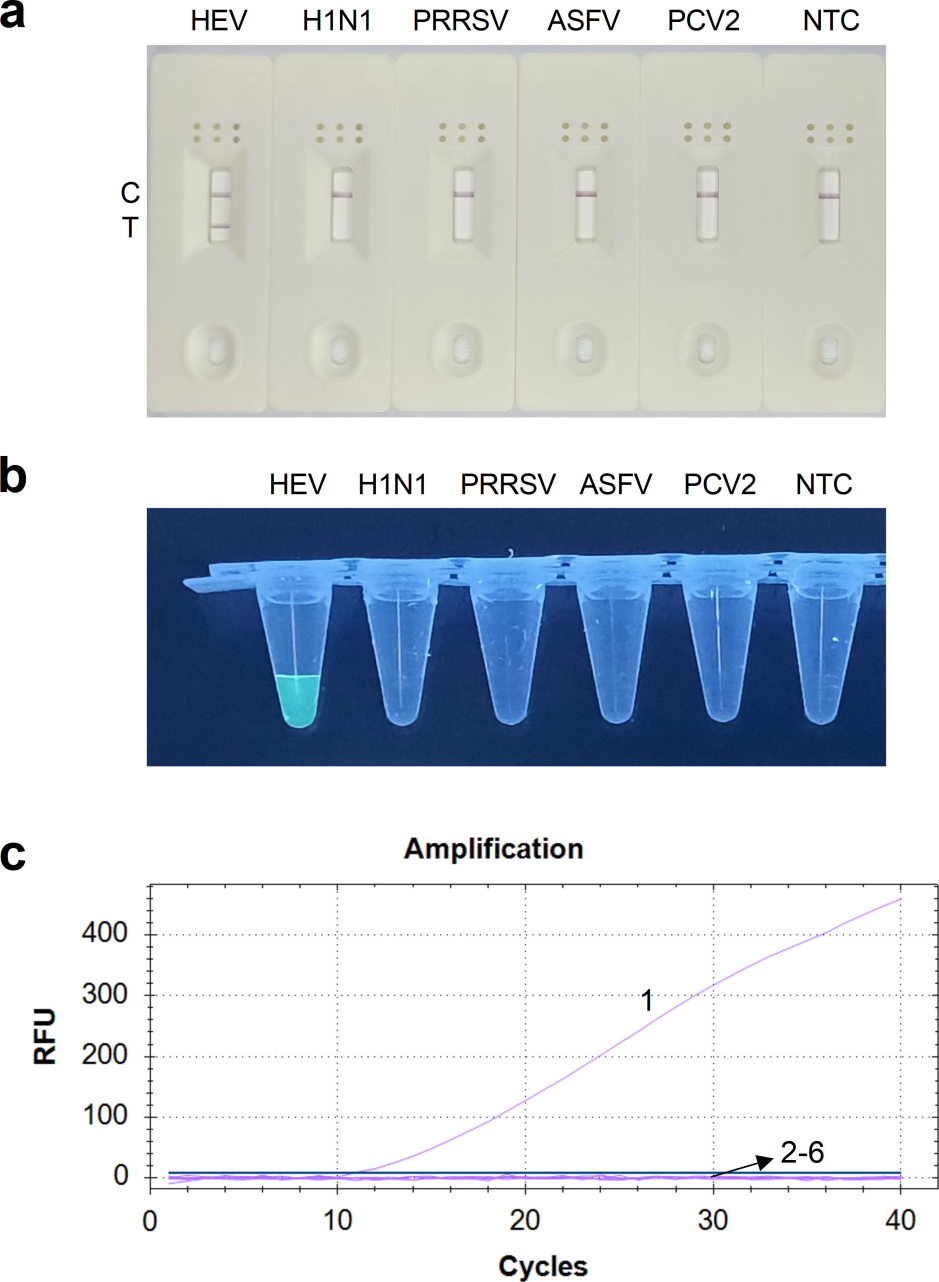
Specificity analysis of the RT-RAA-LFD and qRT-RAA assays for HEV detection. (a) Specificity analysis of the RT-RAA-LFD assay. The amplification products were visualized using LFD. (b–c) Specificity analysis of the qRT-RAA assay. Data collection in the FAM channel occurred every 30 seconds using the real-time PCR instrument. The amplification products were observed under excitation light. Curves 1–6: nucleic acids of HEV, H1N1, PRRSV, ASFV, PCV, and negative control. NTC, no template control.

We used two RT-RAA assays to detect the nucleic acids of HEV and other clinically significant swine pathogens, including H1N1, PRRSV, ASFV, and PCV. The RT-RAA-LFD assay for HEV detection resulted in both the C line and the T line appearing on the LFD, confirming a positive result. Conversely, other clinically important swine viruses and negative control were judged as negative because only the C line appeared (Fig. 4a). As shown in Fig. 4b and c, the qRT-RAA assay for HEV detection showed an obvious amplification curve, with the fluorescence intensity gradually increasing over the reaction time. Upon excitation with incident light, the HEV amplification product emitted green fluorescence, indicating positive detection. In contrast, other viruses and negative control were classified as negative since neither amplification curves nor green fluorescence were detected. Thus, the two RT-RAA assays demonstrated high specificity for HEV detection.

### Sensitivity analysis of the RT-RAA assays

**Fig 5:**
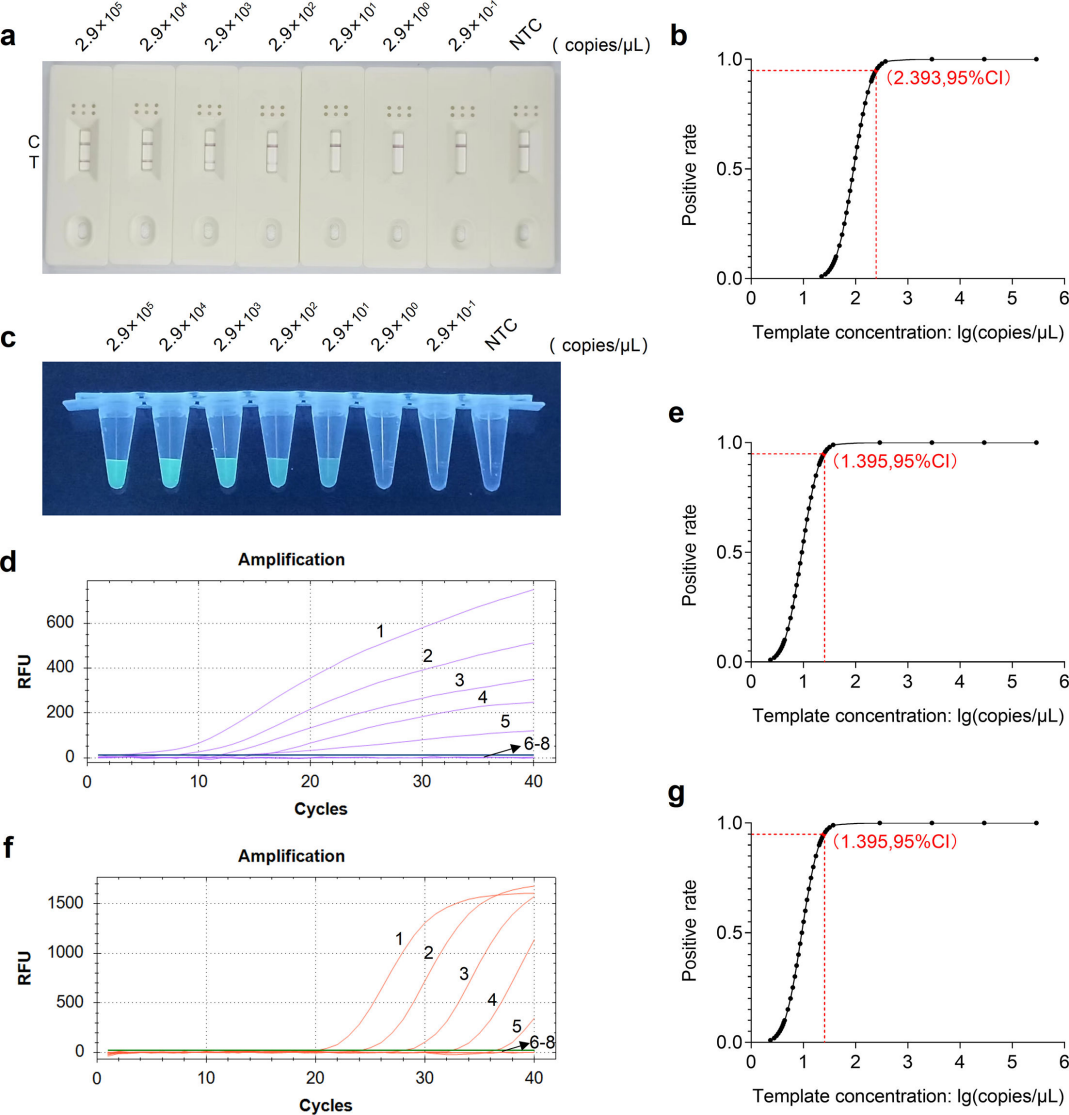
Sensitivity analysis of the RT-RAA-LFD and qRT-RAA and qRT-PCR assays for HEV detection. (a) Sensitivity analysis of the RT-RAA-LFD assay. (b) Probit regression analysis of the RT-RAA-LFD assay. The 95% LOD (247 copies/μL) is depicted by a red dot. (c and d) Sensitivity analysis of the qRT-RAA assay. Curves 1–8: 2.9 × 10^5^–2.9 × 10^−1^ copies/μL of plasmid concentration and negative control, respectively. (e) Probit regression analysis of the qRT-RAA assay. The 95% LOD (25 copies/μL) is depicted by a red dot. (f) Sensitivity analysis of the qRT-PCR assay. Curves 1–8: 2.9 × 10^5^–2.9 × 10^−1^ copies/μL of plasmid concentration and negative control, respectively. (g) Probit regression analysis of the qRT-PCR assay. The 95% LOD (25 copies/μL) is depicted by a red dot. NTC, no template control.

We employed the RT-RAA-LFD, qRT-RAA, and qRT-PCR assays to detect 10-fold serial dilutions of standard plasmids ranging from 2.9 × 10^5^ to 2.9 × 10^−1^ copies/μL. Each dilution was replicated three times to ensure experimental accuracy. The RT-RAA-LFD assay results showed a gradual decrease in the intensity of the T line’s color as the plasmid concentration decreased. The approximate LOD of RT-RAA-LFD was 2.9 × 10^2^ copies/μL (Fig. 5a). In the qRT-RAA assay, as the plasmid concentration decreased, the peak appearance time of the amplification curves gradually lengthened, accompanied by a weakening trend of the green fluorescence intensity. The approximate LOD of qRT-RAA was 2.9 × 10^1^ copies/μL (Fig. 5c and d). Similarly, the qRT-PCR amplification curves exhibited a comparable trend to the qRT-RAA assay. The approximate LOD of qRT-PCR was also 2.9 × 10^1^ copies/μL (Fig. 5f). Probit regression analysis of the detection results from three independent replicate experiments determined that the 95% LOD for the RT-RAA-LFD, qRT-RAA, and qRT-PCR assays were 247 copies/μL (Fig. 5b), 25 copies/μL (Fig. 5e), and 25 copies/μL (Fig. 5g), respectively.

### Analysis of clinical samples using RT-RAA assays

We employed the RT-RAA-LFD, qRT-RAA, and qRT-PCR assays concurrently to detect HEV RNA in 152 bile samples and 93 fecal samples (Table 2). The coincidence rate between RT-RAA-LFD and qRT-PCR across a total of 245 samples was 97.14% (238/245), with a Kappa value of 0.943 (*P* < 0.001) for the consistency test. The coincidence rate between qRT-RAA and qRT-PCR was 98.78% (242/245), with a Kappa value of 0.976 (*P* < 0.001) for the consistency test. These findings indicated that the clinical diagnostic performances of the RT-RAA assays established in this study were comparable to those of qRT-PCR assay.

**TABLE 2:** Clinical diagnostic performance comparison between the RT-RAA-LFD, qRT-RAA, and qRT-PCR assays for HEV detection

Assay			qRT-PCR			Kappa	*P* value	Coincidence rate
			Positive	Negative	Total			
RT-RAA-LFD	Bile	Positive	60	0	60	0.946	＜0.001	97.37%
		Negative	4	88	92			
		Total	64	88	152			
	Feces	Positive	55	0	55	0.932	＜0.001	96.77%
		Negative	3	35	38			
		Total	58	35	93			
qRT-RAA	Bile	Positive	62	0	62	0.973	＜0.001	98.68%
		Negative	2	88	90			
		Total	64	88	152			
	Feces	Positive	57	0	57	0.977	＜0.001	98.92%
		Negative	1	35	36			
		Total	58	35	93			

## DISCUSSION

HEV infection has become a global public health concern due to its potential for cross-species transmission, leading to a rising incidence of foodborne infections in humans. According to the World Health Organization (WHO), HEV causes an estimated 20 million infections annually, which results in 3.3 million symptomatic cases (21). Unfortunately, these cases are often underestimated due to the lack of a reliable gold standard for HEV detection (22). The WHO/FAO JEMRA report highlights the global significance of HEV, emphasizing its zoonotic risk from swine (genotypes 3 and 4) and the public health implications associated with consuming undercooked pork products (23). Economically, HEV infection imposes significant health burdens, including the direct healthcare costs of acute hepatitis and long-term expenses of chronic hepatitis, particularly for immunocompromised persons, organ transplant recipients, those with advanced chronic liver diseases, and pregnant women (24). Currently, Hecolin is the only HEV vaccine licensed for human use, available exclusively in China for the prevention of HEV infection, while no vaccine has been developed for animals (25). The most commonly employed method for HEV detection is TaqMan-based real-time RT-PCR, which relies on a precise and stable thermocycler and a consistent power supply, limiting its practicality in veterinary clinic settings. Therefore, it is crucial to improve existing diagnostic methods and develop new sensitive techniques for better monitoring HEV infection and predicting HEV transmission between domestic animals and humans.

RT-RAA provides an accessible alternative by eliminating the need for expensive variable-temperature equipment, requiring only incubation in a constant-temperature water bath at 37°C to 42°C (26). The amplification products can be detected using compact and lightweight devices such as LFDs, portable thermostatic fluorescence detectors, or portable blue light transilluminators. Therefore, RT-RAA has been widely adopted for detecting various pathogens in both animals and humans (27). Its utility also extends to the detection of pathogens in animal-derived food products (28, 29). In the study, we described a rapid and reliable RT-RAA method, which combines with LFD or real-time fluorescence, for the detection of HEV. By screening the primer-probe pairs of the RT-RAA assays, we demonstrated that the optimal primer-probe has excellent specificity for HEV, without producing false-positive results in detecting other swine pathogens, including H1N1, PRRSV, ASFV, and PCV. We further evaluated the detection performance of the RT-RAA assays. The data showed that our methods can tolerate seven to nine base mismatches at primer and probe binding sites when the HEV-3 KernowC1/p6 strain was used as a template, without significantly affecting the performance of RT-RAA analysis. This result is similar to previously reported RT-RPA primers and probes, which can tolerate four to nine nucleotide mismatches (30). Moreover, these RT-RAA assays were validated for their high sensitivity in detecting HEV RNA in swine bile samples, with a significant reduced detection time compared with traditional methods such as RT-nPCR and qRT-PCR.

Domestic pigs are considered the main natural reservoir for HEV-3 and HEV-4 genotypes (31). HEV RNA can be detected in pork livers, bile, and fecal samples. A study conducted in Spain reported that among 69 pigs with different pathological conditions, HEV RNA was detected in 26 pigs using nested PCR. They found that bile was the most frequently HEV-positive sample (13/69), followed by liver (10/69) (32). Another study investigated on family-owned farms in Brazil found that HEV RNA was detected in 8 out of 25 pigs via nested PCR, with bile as the most commonly detected sample (7/25), followed by liver tissue (6/25) (33). These studies indicated a higher likelihood of HEV presence in bile compared with liver during the infection stage. Using nested PCR, HEV RNA was detected in 0.23% of market samples (liver, pork, and intestinal samples) and 3.93% of slaughterhouse samples (bile and fecal samples) in Bangkok fresh markets and slaughterhouses (34). At a slaughterhouse in Northwestern Italy, 156 pigs were sampled, with HEV RNA detected in 21 pigs through real-time quantitative PCR, showing the highest positive rate in feces (19/156), followed by bile (13/156) and liver tissue (8/156) (35). In the present study, analysis of clinical samples, including bile and fecal specimens from slaughtered swine, demonstrated high concordance between both RT-RAA assays and qPCR, with 97.14% for RT-RAA-LFD and 98.78% for qRT-RAA. These findings highlight the diagnostic tool holds great potential for accurate and rapid screening of swine HEV infections.

Most of the HEV strains that infect humans and other mammals belong to Orthohepevirus A, which has eight genotypes (HEV-1–HEV-8). To address potential nucleotide sequence overlaps among different HEV genotypes, we downloaded and aligned 45 genomic sequences of HEV-1 and HEV-2 strains (Fig. S3a), 5 genomic sequences of HEV-5 and HEV-6 strains (Fig. S3b), and 9 genomic sequences of HEV-7 and HEV-8 (Fig. S3c) from the GenBank nucleic acid sequence database on NCBI. Our comparative analysis revealed that the consensus sequence between HEV-1 and HEV-2 or HEV-7 and HEV-8 shared a high degree of similarity with the corresponding nucleotide fragments in HEV-3 and HEV-4. This suggested that our primers and probes are likely suitable for detecting HEV-1, HEV-2, HEV-7, and HEV-8. However, we identified more than nine mismatched bases at the binding sites of the final primer pair and exo-probe with the consensus sequence between HEV-5 and HEV-6, which may affect the performance of qRT-RAA analysis. Therefore, further validation studies are required to evaluate the effectiveness of these primers and probes across all HEV genotypes mentioned.

However, the RT-RAA assays established in this study still require improvements. The current methodologies involve total RNA extraction using TRIzol reagent, a process that is time-consuming and cumbersome. Thus, there is a pressing need to develop a rapid and simplified sample preparation method to enhance the efficiency and convenience of the RT-RAA assays. Several RNA/DNA extraction-free RPA/RAA assays have already been employed in clinical diagnostics, promoting the development of rapid detection methods (36–38).

In conclusion, we described two HEV ORF2-based rapid, reliable, and cost-effectiveness RT-RAA assays, including the RT-RAA-LFD and qRT-RAA assays. Both assays demonstrated excellent performance in detecting HEV in swine bile and fecal samples. These tools hold significant potential for HEV screening in animal-derived food, particularly in laboratories with limited equipment or resource-constrained settings, thereby contributing to animal-derived food safety. Eliminating the infected animals from the food chain would substantially reduce the risk of HEV transmission to consumers. Additionally, optimizing RNA extraction steps and addressing cost-related challenges will improve the efficiency and accessibility of RT-RAA assays, further solidifying their role in veterinary clinical HEV detection.

## SUPPLEMENTAL MATERIAL

Supplemental figures jcm.01064-24-s0001.docx Figures S1 to S3.

ASM does not own the copyrights to Supplemental Material that may be linked to, or accessed through, an article. The authors have granted ASM a non-exclusive, world-wide license to publish the Supplemental Material files. Please contact the corresponding author directly for reuse.
