# Supplementary material for: Rapid visual detection of hepatitis E virus combining reverse transcription recombinase-aided amplification with lateral flow dipstick and real-time fluorescence
Source: J Clin Microbiol. 2025 Jan 16;63(2):e01064-24. doi: 10.1128/jcm.01064-24 (PMC11837526; doi:10.1128/jcm.01064-24)
Supplement: Supplemental figures — Figures S1 to S3. [file jcm.01064-24-s0001.docx]

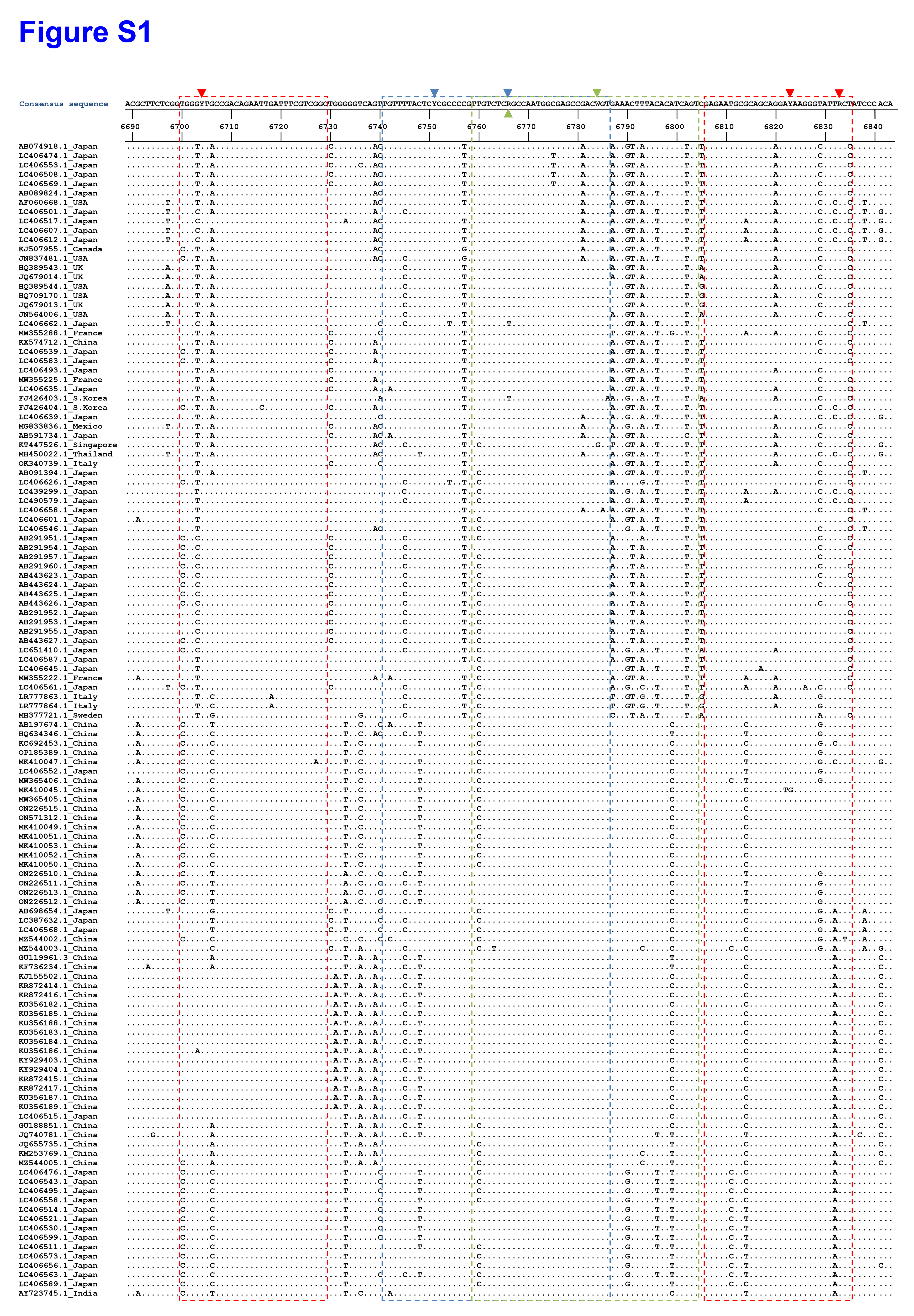
**Figure S1.** The positions of the final primer pair, nfo-probe and exo-probe in ORF2 gene of HEV-3 and HEV-4. The GenBank accession numbers and the respective countries or regions of origin for various HEV strains are shown on the left side of the figure. The dots signify highly conserved nucleotide residues. The red dashed boxes show the positions of the upstream and downstream primers within the sequence. The blue and green dashed boxes show the positions of the nfo-probe and the exo-probe, respectively. Furthermore, the red arrows indicate the incorporation of degenerate bases Y and R in the design of the upstream and downstream primers. Similarly, the blue and green arrows indicate the incorporation of degenerate bases Y, R, and W in the design of the nfo-probe and the exo-probe, respectively. Here, Y represents either cytosine (C) or thymine (T), R represents either adenine (A) or guanine (G), and W represents either adenine (A) or thymine (T).


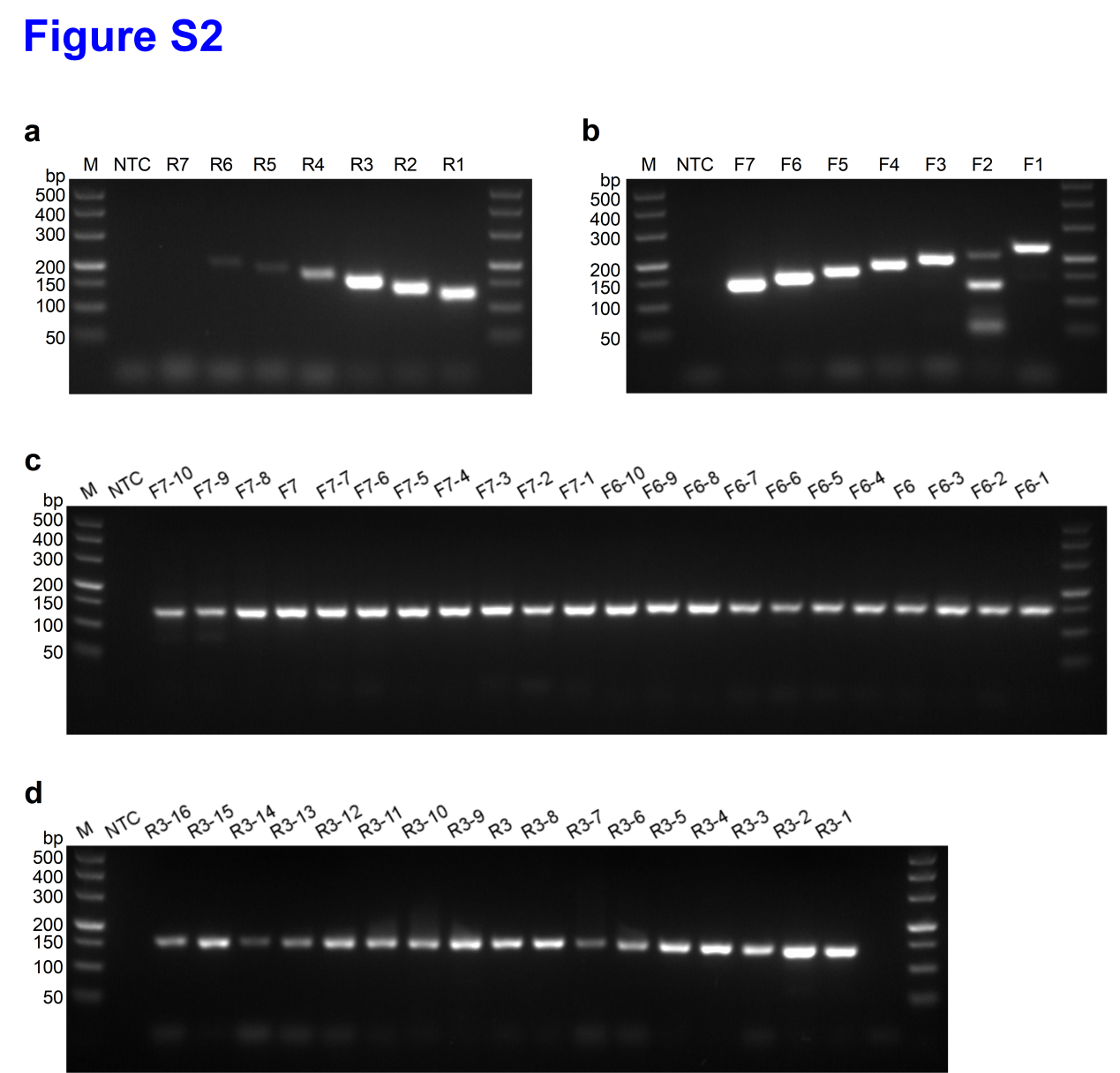


**Figure S2**. Screening of the optimal primer pair for the RT-RAA assays shown in more comprehensive agarose gel images. (a) Primary screening results analyzed by agarose gel electrophoresis for the optimal reverse primer. (b) Primary screening results analyzed by agarose gel electrophoresis for the optimal forward primer. (c) Secondary screening results analyzed by agarose gel electrophoresis for the optimal forward primer. (d) Secondary screening results analyzed by agarose gel electrophoresis for the optimal reverse primer. NTC, No template control; M, DL500 DNA Marker.


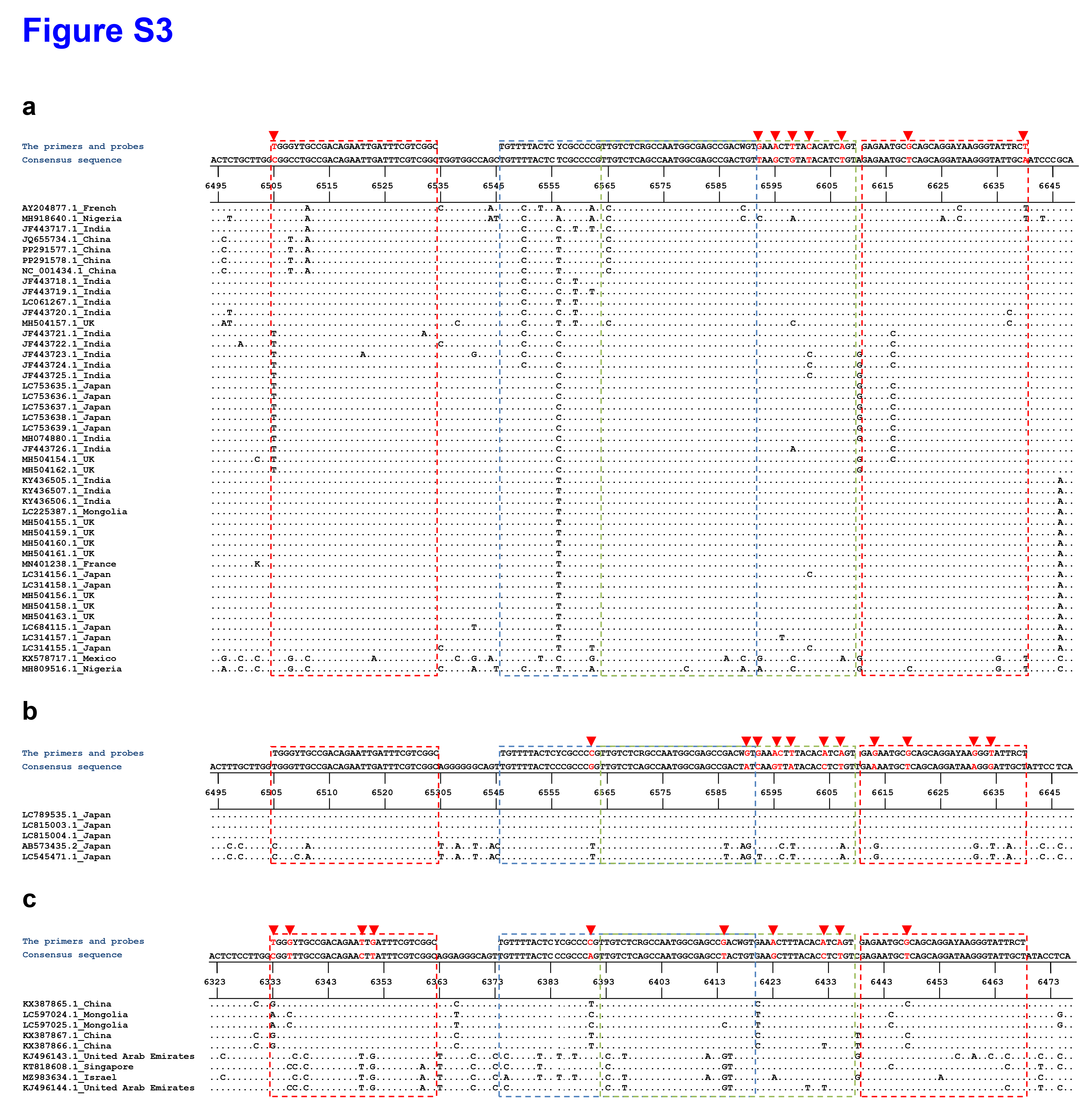


**Figure S3.** The positions of the final primer pair, nfo-probe and exo-probe in ORF2 gene of HEV across different genotypes. (a) The positions of the final primer pair, nfo-probe and exo-probe in ORF2 gene of HEV-1 and HEV-2. The red arrows indicate the mismatched bases at the binding sites of primers and probes with the consensus sequence between HEV-1 and HEV-2. (b) The positions of the final primer pair, nfo-probe and exo-probe in ORF2 gene of HEV-5 and HEV-6. The red arrows indicate the mismatched bases at the binding sites of primers and probes with the consensus sequence between HEV-5 and HEV-6. (c) The positions of the final primer pair, nfo-probe and exo-probe in ORF2 gene of HEV-7 and HEV-8. The red arrows indicate the mismatched bases at the binding sites of primers and probes with the consensus sequence between HEV-7 and HEV-8.
